# Prostatic urethral lift for subjects in urinary retention (PULSAR): 12‐Month results of a prospective controlled trial compared with real‐world outcomes

**DOI:** 10.1002/bco2.280

**Published:** 2023-09-08

**Authors:** Mark Rochester, Ruth Doherty, Toby Page, Neil Barber, Rajesh Kavia, Nikesh Thiruchelvam, Steven Gange, Thomas Mueller, Gregg Eure, Peter Chin, Oliver Kayes

**Affiliations:** ^1^ Norfolk and Norwich University Hospital Norwich UK; ^2^ Freeman Hospital Newcastle upon Tyne UK; ^3^ Frimley Park Hospital Frimley UK; ^4^ Northwick Park Hospital Harrow UK; ^5^ Cambridge University Hospitals NHS Foundation Trust Cambridge UK; ^6^ Summit Urology Group Murray Utah USA; ^7^ Delaware Valley Urology Voorhees New Jersey USA; ^8^ Urology of Virginia Virginia Beach Virginia USA; ^9^ South Coast Urology Wollongong New South Wales Australia; ^10^ Leeds Teaching Hospitals and University of Leeds Leeds UK

**Keywords:** acute urinary retention, benign prostatic hypertrophy, bladder outflow obstruction, bladder voiding efficiency, prostatic urethral lift

## Abstract

**Objective:**

To examine the safety and efficacy of prostatic urethral lift (PUL) in acute urinary retention (AUR) patients within a controlled (PULSAR) and real‐world setting (Real‐World Retrospective study).

**Materials and methods:**

PULSAR was a 12‐month prospective study of PUL in AUR patients (*n* = 51) performed at six centres in the United Kingdom; enrolled BPH patients aged ≥50 years, with prostate volume of ≤100 cc. AUR was defined as being catheter dependent with at least one prior failed trial without catheter (TWOC) while on an alpha‐blocker. RWR consisted of 3226 consecutive PUL patients across 22 international sites treated between July 2017 and March 2020; 469 of whom were in urinary retention (RWRr), that is, catheter‐dependent at the time of their procedure. Symptom response, uroflow and catheter independence rates were compared between PULSAR and RWRr subjects. A logistical regression model was constructed to evaluate patient baseline and dynamic factors predicting success after the procedure.

**Results:**

Seventy‐three percent of PULSAR subjects were catheter independent and free from surgical reintervention at 12 months post‐PUL. Success was associated with higher voiding efficiency during the perioperative period. Slightly higher catheter‐independent rates (80%) were seen in RWRr patients; variables that influenced success included age <70 years, lower baseline prostate‐specific antigen (PSA), lower baseline post‐void residual (PVR) and shorter pre‐procedural catheter duration. Logistic regression of the combined PULSAR and RWRr retention groups revealed that procedural age <70 years and higher bladder voiding efficiency (BVE) were associated with success.

**Conclusions:**

Lower baseline PSA and PVR, younger age and shorter pre‐procedure catheter durations drove successful outcomes in AUR patients undergoing PUL. Post‐PUL voiding efficiencies may help ascertain long‐term response to treatment.

## 1 INTRODUCTION

Benign prostatic hyperplasia (BPH) is a progressive quality‐of‐life disease that, when left untreated, can lead to acute urinary retention (AUR) and bladder function deterioration. BPH‐related problems place significant burdens on healthcare, as it has been shown that some men who suffer an AUR event have significantly poorer outcomes with increased mortality.^1^ Therefore, expedient assessment and treatment may be critical to improving overall outcomes.

Over half of AUR cases are attributed to progressive BPH; as lower urinary tract symptoms (LUTS) worsen, patients requiring intervention are at risk for AUR.^2^ Initial episodes of AUR are treated with catheterization and alpha‐blocker initiation; subsequent voiding trials are likely to fail in up to 50% of patients.^3^ In men who fail their voiding trial, long‐term catheterization is associated with risks of infection, multiple interactions with healthcare providers, and reduction of the patient's quality of life (QoL).^4^ As such, some men may opt for a traditional resection surgery such as transurethral resection of the prostate (TURP), photovaporization of the prostate (PVP), or holmium laser enucleation of the prostate (HoLEP).^5^ Though these surgical options are known to be effective in treating LUTS, complications can include infection, bleeding, pain, incontinence (stress and/or long‐term urinary urge incontinence) and sexual dysfunction. Furthermore, full recovery may be prolonged in this group of patients.^6^

As LUTS‐BPH treatment delays can potentially lead to episodes of AUR,^7^ it is imperative for urologists to have safe and effective treatments that can be administered in a timely manner. Emerging minimally invasive surgical treatments (MIST) may fulfil this role. To date, the prostatic urethral lift (PUL) procedure utilizing the UroLift System is the most widely adopted BPH MIST within the National Health Service (NHS) in the United Kingdom.^8^ PUL uses transprostatic implants to relieve symptoms of urinary outflow obstruction without cutting, heating or removing tissue using an energy source. PUL can be used to treat a wide range of prostate sizes and anatomies up to 100 cc; furthermore, it can be routinely performed as a day case procedure under sedation or local anaesthesia.^9^ Some UK centres have even moved to an ambulatory setting outside of a traditional day (or inpatient) surgical setting thereby foregoing the need for anaesthetic support.^10^, ^11^

PUL randomized studies have demonstrated rapid and significant improvement in LUTS with durability through 5 years. Recovery often occurs within days or weeks, and typically, in non‐retention cases, no post‐operative catheter is placed. Adverse events are commonly mild to moderate, resolving within 2 weeks; similarly, PUL is not reported to cause any de novo and sustained sexual dysfunction.^12^ Additional benefits include shorter operative times and reduced hospital stay compared with traditional surgery. These hallmarks of PUL have been well‐documented in LUTS‐BPH patients with established voiding, albeit with obstructed uroflowmetric parameters. Proven gains relate to positive outcomes affecting both patient care and healthcare systems; these include increased efficacy, safety, efficiency and cost–benefit improvements.^9^ Insofar as PUL is effective in treating AUR, adopting PUL into the clinical pathway for men with AUR should provide tangible quality improvements aligned to the principles set by the UK NHS Getting It Right First Time (GIRFT) initiative.^13^ These measurable benefits can include reduced waiting times, shorter care pathways, reduced burden on inpatient surgical resources (staff, beds, operating theatres), fewer complications (dysuria, haematuria, pelvic pain/discomfort) and shorter overall catheterization times. Here, we present the results of the PULSAR study, a prospective single‐arm trial conducted across six sites in the United Kingdom, which was undertaken to determine the safety and efficacy of PUL for AUR patients through 12 months post‐treatment. Outcomes of PULSAR subjects are compared with urinary retention patients from the large real‐world retrospective (RWRr) study of PUL as well as PUL subjects from the LIFT pivotal trial.

## 2 MATERIALS AND METHODS

### 2.1 Study protocols of participants used in comparative analyses

#### 2.1.1 PULSAR study

A prospective feasibility study of PUL in AUR patients was performed in six study centres in the United Kingdom following HRA and IRAS approval (REC reference 17/WA/0242). Study enrolment occurred between March 2018 and June 2019; the study period was 12 months. Enrolment criteria included LUTS‐BPH patients age ≥ 50 years, prostate volume ≤ 100 cc as measured by transrectal or transabdominal ultrasound (US) and AUR with at least one failed trial without catheter (TWOC) while on alpha‐blocker. Excluded from the study were men with an obstructive or protruding median lobe, men who had undergone prior surgical intervention for BPH, a chronic retention volume of >1500 mL and those with a history of bladder or prostate cancer, neurogenic or atonic bladder.

#### 2.1.2 LIFT trial

The pivotal randomized controlled trial of PUL (i.e., the LIFT trial) included 206 men (randomized to either PUL or sham) who met the following enrolment criteria: ≥50 years of age, IPSS ≥ 13, Qmax ≤ 12 mL/s and prostate volumes 30–80 cc. Notable exclusions included subjects with prior surgical treatment for BPH, an active urinary tract infection, median lobe obstruction and urinary retention. Subjects randomized to PUL were followed for 5 years post‐treatment.

#### 2.1.3 Real‐world retrospective (RWR) study of PUL

The large RWR study of PUL included 3226 patients across 22 international sites in the United States, Australia and the United Kingdom treated with PUL following market clearance. Sites were initiated, and chart reviews were conducted between July 2017 and March 2020. Included in the database were consecutive PUL patients that had a baseline IPSS score and at least one IPSS score within 12 months post‐treatment. Patients in urinary retention at the time of their procedure did not require a baseline IPSS, and retention status was confirmed through medical history records and catheterization logs confirming the presence of a urinary catheter at day zero. RWR data were filtered into non‐retention (*n* = 2714) and retention groups (RWRr, *n* = 512).

### 2.2 The PUL procedure

The PUL procedure is performed by deploying small transprostatic UroLift implants under cystoscopic guidance to mechanically retract the obstructing prostatic lobes. A delivery device (the UroLift® System, Neotract Inc, Teleflex, Pleasanton, CA) accesses the prostatic urethra, compresses the obstructive lobe and deploys the implant through the lobes of the prostate. The implant consists of a nitinol capsular table (CT) and stainless‐steel urethral end‐piece (UE) joined by a monofilament PET suture. During the procedure, a 19‐gauge needle housing the implant is deployed through the prostatic lobe and capsule. Upon needle retraction, the CT deploys and engages the prostatic capsule, and the suture is tensioned and secured in place by the deployment of the UE. Additional implants are delivered as required. Investigators for the PULSAR study were required to have performed a minimum of 30 prior PUL procedures.

### 2.3 PULSAR study endpoints

PULSAR subjects were followed at 3 days, 6 weeks and 3, 6 and 12 months post‐PUL. The primary endpoint was successful TWOC rate during the perioperative period. Success was defined as voiding spontaneously (with a recorded volume of ≥100 mL) and a post‐void residual volume of <300 mL (as measured by ultrasound) at 3 days (±1 day) post‐index procedure. Subjects who failed the initial TWOC were monitored for voiding ability in subsequent follow‐up visits; catheter‐free rates were also assessed at one and 12 months. The primary safety assessment was the incidence of serious adverse events (SAEs) through 3 months post‐PUL. IPSS, QoL, Qmax, PVR and Sexual Health Inventory for Men (SHIM) score were collected at each timepoint when available. Urodynamic parameters including detrusor pressure at maximum flow rate (PdetQmax) and the bladder outlet obstruction index (BOOI) when obtained were compared at baseline and at 12 months. Non‐SAE rates as well as surgical retreatment rates were calculated through 12 months post‐PUL.

### 2.4 Statistics

The PULSAR primary safety and efficacy endpoints were analysed on an intent‐to‐treat (ITT) basis. Additional analyses were conducted on a per‐protocol basis. Subjects who underwent surgical retreatment with either PUL, TURP or HoLEP were censored the day after their surgical retreatment.

To evaluate patient baseline and dynamic factors associated with successful outcomes following PUL, logistical regression models were constructed incorporating the PULSAR and RWRr cohorts. A successful response following PUL was defined as catheter‐free at 12 months post‐treatment and no secondary BPH surgical event. Results were reported as odds ratio (OR) point estimates with statistical significance (*p*‐values) quantified using chi‐squared tests. All statistical analyses were performed using SAS software version 9.4 (SAS Institute, Inc.).

#### 2.4.1 Comparative analyses utilizing LIFT and RWR

PULSAR outcomes including IPSS, QoL, Qmax and SHIM were compared with LIFT through 12 months post‐PUL. The RWRr cohort was filtered to exclude patients that had prior BPH surgery; IPSS, QoL, Qmax, PVR, catheter‐free rates and adverse events rates were compared between RWRr and PULSAR through 12 months. Mean change and percentage change from baseline was compared using 95% confidence intervals and paired *t*‐tests; absolute symptom scores were compared between groups using unpaired *t*‐test. Catheterization and adverse event rates were analysed using chi‐squared and, when appropriate, Fisher's exact test. Analysed cohorts were statistically powered to permit comparative analysis.

## 3 RESULTS

### 3.1 PULSAR study outcomes

**FIGURE 1:**
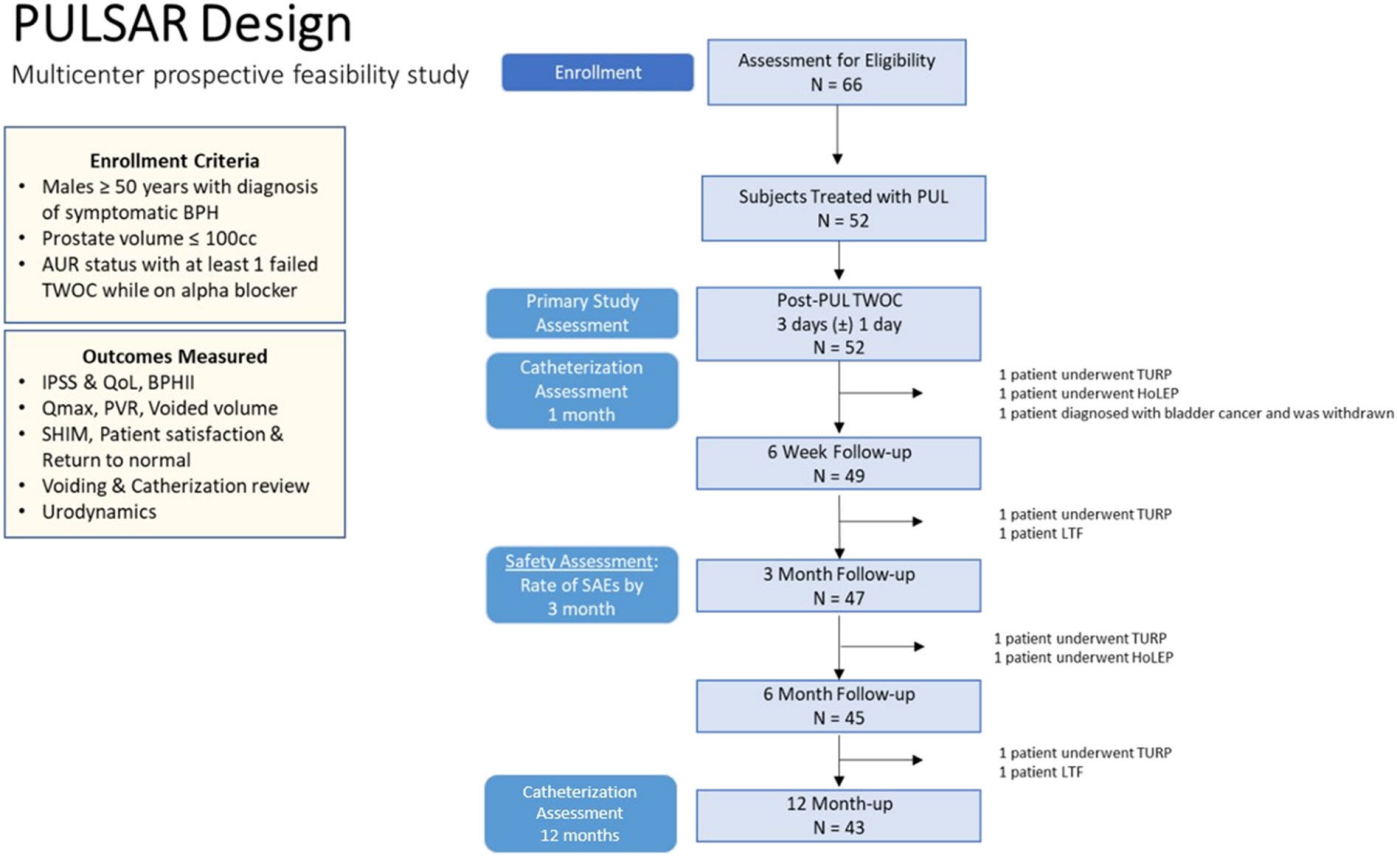
Design of the prostatic urethral lift for subjects in acute urinary retention (PULSAR) study.

All 52 enrolled subjects were treated with PUL and were available for ITT primary endpoint analyses; 43 (81%) completed all 12 months of follow‐up (Figure 1). PULSAR subjects were on average 71 years old (range 53–86) with 40%, 37% and 23% of patients enduring LUTS for ≥6 years, 1–5 years and <1 year prior to PUL, respectively (Table 1). Mean duration of urinary retention prior to PUL was 132 ± 112 days. Overall procedural time was 31.3 ± 12.1 min; an average of 4.8 ± 1.36 implants were placed.

**TABLE 1:** Demographics, intraoperative and postoperative.

Mean, SD [Range] (*N*)	PULSAR	LIFT	RWR retention
Pre‐op characteristics			
Age	71.3, 7.9 [53–86], (52)	67, 8.6 [49–86], (140)	70.8, 10.7 [36–94], (469)
Prostate volume (cc)	55.17, 22.84 [10.0–98.6], (52)	44.54, 12.47 [30.0–77.1], (140)	48.5, 45.9 [14–130], (211)
Days in retention	132, 112 [15–660], (52)	‐‐	156.3, 357.9 [1–3167], (324)
Baseline BPH medication duration (days)			
Alpha‐blocker	877.8, 1413.93 [9–5077], (52)	‐‐	455.9, 528.1 [3–2834], (311)
5ARI	525.9, 589.66 [26–2034], (15)	‐‐	417.5, 525.5 [3–2534], (96)
Combination	509.0, NA [509–509], (1)	‐‐	391.5, 58.7 [350–433], (2)
Sexual Function			
SHIM total score	16.0, 8.7 [2–25], (22)	15.95, 6.94 [2.0–24.0], (107)	‐‐
Intraoperative data			
Devices implanted	4.8, 1.36 [2–7], (52)	4.9, 1.6 [2–11] 140	4.6, 1.4 [2–9], (469)

After PUL, the mean post‐operative hospitalization time was approximately 2 h (0.08 days). Per the study protocol, all subjects were catheterized prophylactically after PUL. At day 3, 56% (*n* = 29) of PULSAR subjects completed a successful TWOC. The catheter independence rate increased throughout the duration of the study, improving to 71% (*n* = 37) at 1 month and 73% (*n* = 38) at 12 months. Eight subjects required intermittent self‐catheterization (ISC) after the removal of their Foley catheter, five of whom achieved catheter independence by 12 months. The remaining subjects continued ISC (*n* = 2) or required a surgical retreatment (*n* = 1).

Mean return to pre‐operative activity for PULSAR subjects was 8.5 days. At each follow‐up visit, subjects rated their satisfaction with their LUTS‐BPH symptoms, revealing high levels of satisfaction. At 12 months, 80.5% of subjects reported being ‘very much better or much better’, and 87.8% stated that they would recommend the procedure to a friend or relative. Forty‐two percent of subjects were sexually active at baseline with SHIM scores of 16 ± 8.7 that increased to 17.2 ± 7.6 at 6 weeks and 19.3 ± 7.8 at 3 months and remained stable at 19.3 ± 7 at 12 months (Table 2).

**TABLE 2:** Unpaired outcome measures after PUL—6 weeks to 12 months in PULSAR.

Test Mean, median, SD [range], (*N*)	Baseline	Timepoint			
		6 weeks	3 months	6 months	12 months
IPSS total score	‐‐	10.5, 10.0, 7.8 [0–26], (41)	9.1, 8.0, 6.8 [0–24], (44)	9.6, 9.0, 7.0 [0–28], (45)	10.3, 8.0, 7.4 [0–26], (45)
QoL	‐‐	1.6, 1.0, 1.4 [0–5], (41)	1.3, 1.0, 1.6 [0–5], (44)	1.5, 1.0, 1.5 [0–5], (45)	1.5, 1.0, 1.5 [0–5], (45)
BPHII	‐‐	2.3, 1.0, 2.6 [0–9], (41)	1.6, 0.0, 2.6 [0–12], (44)	1.6, 0.0, 2.5 [0–9], (45)	2.2, 1.0, 3.0 [0–12], (45)
Qmax (mL/s)	‐‐	10.9, 9.3, 10.0 [1–64], (48)	10.4, 9.7, 5.7 [1–33], (50)	‐‐	10.9, 8.9, 8.5 [1–42], (50)
PVR (mL)	‐‐	162.4, 111.5, 177.6 [0–800], (50)	160.8, 116.0, 169.8 [0–800], (51)	‐‐	154.1, 120.0, 167.7 [0–800], (51)
SHIM	16.0, 20.5, 8.7 [2–25], (22)	17.2, 21.0, 7.6 [4–25], (9)	19.3, 23.5, 7.8 [5–25], (12)	‐‐	19.3, 22.5, 7.0 [2–25], (14)

Urodynamic testing was performed at baseline (i.e., within 30 days of index procedure) and at 12 months post‐PUL; the urodynamic traces were analysed and reported centrally by a single expert (RD). As urodynamics are not commonly conducted in BPH trials because of the invasiveness of procedure, the assessment was curtailed to 12 willing subjects at a single site. Within this subset of patients, mean detrusor pressure at max flow (PdetQmax) decreased by 15.0 ± 11.7, representing a 23% improvement from baseline. Concurrently, a decrease of 18.2 ± 24 in the BOOI was recorded, reflecting a 41% improvement from baseline. Five subjects moved out of the obstructed zone as measured by the ICS nomogram; of those who remained in the obstructed or equivocal zone, 86% were catheter‐free and 83% felt ‘very much better’.

Adverse events were assessed as treatment‐related and were assigned Clavien–Dindo grades. For the primary safety assessment through 3 months, a total of four SAEs occurred (Clavien–Dindo grade IIIa), all of which were described as haematuria with clot retention within 5 days of the procedure and resolving after an average of 2 days. Most adverse events were non‐serious, mild to moderate (Clavien–Dindo grade I & II), with more than half (57.4%) resolving within 4 weeks, and 78.7% resolving within 3 months. Of these mild to moderate/Clavien–Dindo grade I and II events, haematuria and dysuria were the most commonly reported.

Over the course of the 12‐month follow‐up, eight patients (15.8%) underwent surgical retreatment with TURP (*n* = 4), HoLEP (*n* = 2) or PUL (*n* = 2). The majority of patients (40/52, 77%) discontinued their LUTS medications prior to or on the day of the PUL procedure. Most of these patients (38/52, 73%) remained medication‐free at study end, with only two subjects restarting their medical therapy during follow‐up.

### 3.2 PULSAR symptom outcomes compared with LIFT and RWR

Because of their urinary retention status, PULSAR subjects lacked baseline IPSS, QoL and Qmax values; however, absolute symptom scores were collected at all timepoints following treatment. At 6 weeks post‐PUL, IPSS, QoL and Qmax absolute scores averaged 10.5 ± 7.8, 1.6 ± 1.4 and 10.9 ± 8.5, respectively. Throughout follow‐up, urinary symptom scores remained stable. At 12 months, mean IPSS, QoL and Qmax were as follows: 10.3 ± 7.4, 1.5 ± 1.5 and 10.9 ± 8.5 (Table 2).

**FIGURE 2:**
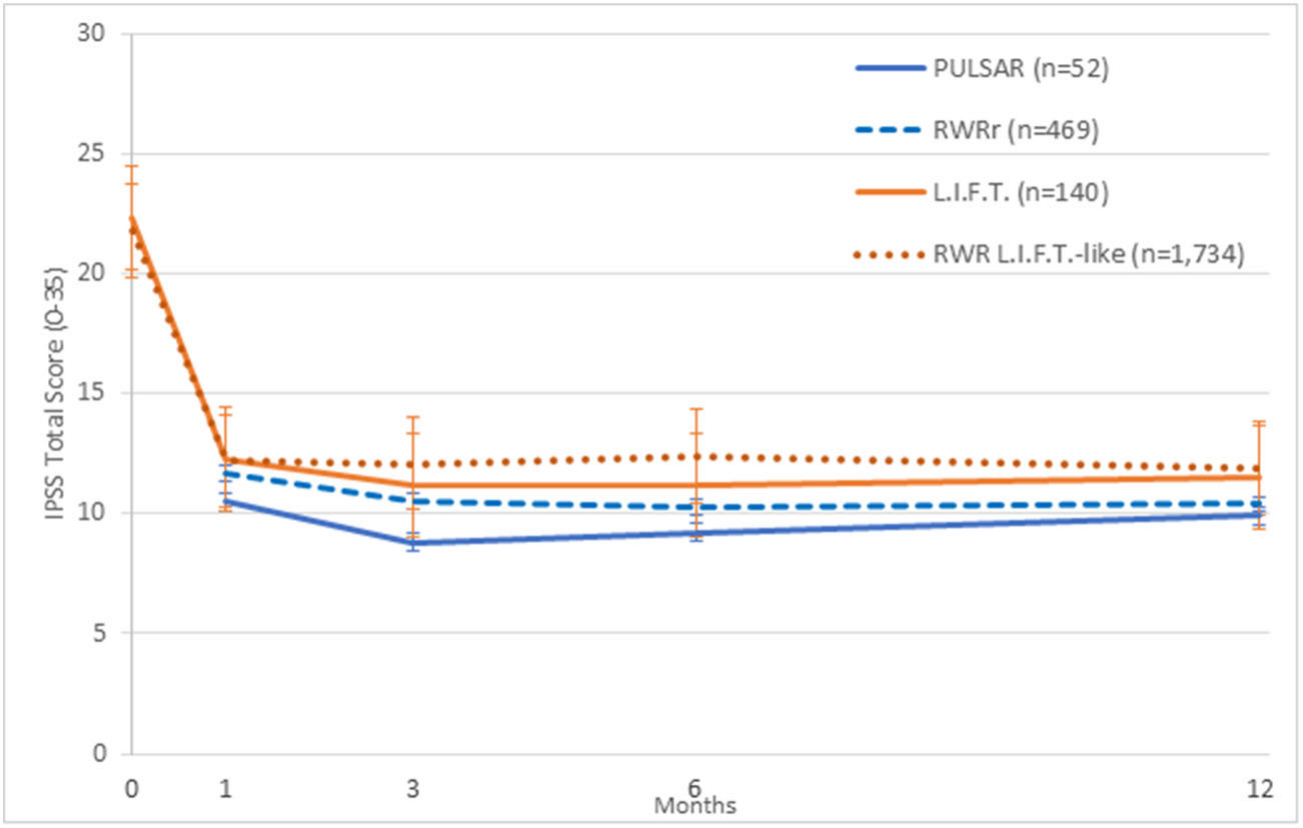
IPSS outcomes for urinary retention and spontaneously voiding patients in real‐world and controlled trial settings (PULSAR, LIFT, RWR retention, RWR non‐retention) are consistent through 12 months.

Compared with LIFT subjects, PULSAR subjects were older (71 vs. 67 years, *p* < 0.01) with larger prostates (55 vs. 45 cc, *p* < 0.01). Despite these differences, absolute IPSS and Qmax scores were similar between PULSAR and LIFT patients at 12 months post‐treatment (11.5 vs. 9.9, *p* = 0.23; 12.1 vs. 11.7, *p* = 0.75; Figure 2). Absolute QoL scores, however, were significantly better for PULSAR subjects at all timepoints (12‐month QoL PULSAR vs. LIFT: 1.4 vs. 2.3, *p* < 0.01). In both LIFT and PULSAR studies, erectile function was preserved, with no differences in absolute SHIM scores through 12 months post‐PUL.

Within the large RWR study of PUL, 512 subjects were in urinary retention at the time of their procedure, 469 of whom did not have a prior BPH surgery and were used as a real‐world comparator (RWRr) to PULSAR. Preoperative characteristics (age; time from first retention episode to PUL), baseline BPH medication use and the number of implants placed were largely consistent between both groups, though PULSAR subjects had larger prostate volumes at baseline (55 vs. 48 cc, *p* = 0.04; Table 1). After PUL, RWRr patients experienced similar catheter‐free rates at 30 days (71% vs. 72%, *p* = 0.9) and 12 months (73% vs. 80%, *p* = 0.2) compared with PULSAR subjects. Absolute IPSS and Qmax scores at 12 months (IPSS: 9.9 vs. 10.4, *p* = 0.73; Qmax: 11.7 vs. 13.1, *p* = 0.48) were also similar between studies; PULSAR subjects reached better absolute QoL scores at all timepoints through 12 months (12‐month QoL PULSAR vs. RWRr: 1.4 vs. 2.3, *p* = 0.02). Overall adverse event rates for RWRr patients were not elevated compared with PULSAR subjects (34.1% vs. 46.2%, *p* = 0.09), and 47 RWRr patients underwent a surgical retreatment within 12 months of the PUL procedure. The average follow‐up length for the RWRr group was 287 days.

### 3.3 Successful PUL response in AUR subjects

The definition of a successful response following PUL (i.e., a responder) was remaining catheter‐free at 12 months post‐treatment and free from a secondary surgical retreatment event. For PULSAR subjects, higher bladder voiding efficiency (BVE), that is, the ratio of the voided volume to the total bladder capacity, at day three following PUL was associated with success (mean responder BVE, 54; mean failure voiding efficiency, 29; OR: 1.02, *p* < 0.05). In the RWRr patients, variables that influenced success included younger procedural age < 70 years (mean responder age, 69.5; mean failure age, 72.3; OR: 1.78, *p* < 0.05), lower baseline PVR (mean responder PVR, 228.9; mean failure PVR, 504.4; OR: 1.002, *p* < 0.01), lower baseline PSA (mean responder PSA, 3.1; mean failure PSA, 5.8; OR: 1.06, *p* < 0.05) and shorter pre‐procedural catheter duration (mean responder duration, 43.5 days; mean failure duration, 204.2 days; OR: 1.004, *p* < 0.01) (Table 3).

**TABLE 3:** Univariate analysis of patient and procedural characteristics predictive of resolution of retention following PUL.

Covariate	Responder mean	Failure mean	Odds ratio	*P*‐value
PULSAR				
Voiding efficiency	54	29	1.02	<0.05
RWR retention (RWRr)				
Age <70	69.5	72.3	1.78	<0.05
PSA	3.1	5.8	1.06	<0.05
PVR	228.9	504.4	1.00	<0.01
Pre‐procedure catheter (duration in days)	43.5	204.2	1.00	<0.01
Combined (PULSAR & RWRr)				
Age <70	69.7	72.7	2.04	<0.01
Voiding efficiency	59.0	32.0	1.02	0.02
Pre‐procedure catheter (duration in days)	53.2	198.7	1.00	<0.01

**FIGURE 3:**
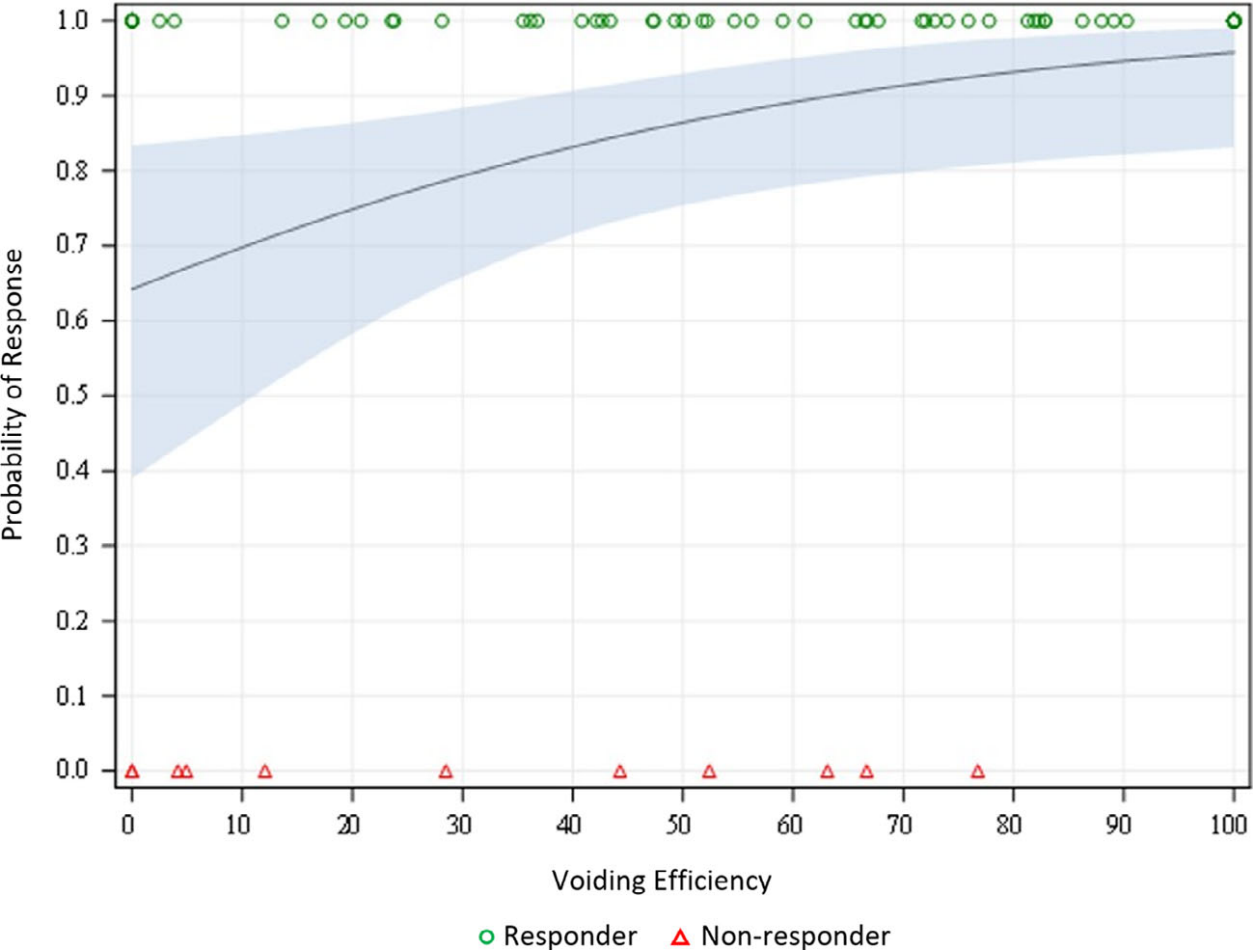
Perioperative bladder voiding efficiency measurement predicts success in retention patients following PUL.

Logistic regression on combined PULSAR and RWRr subjects revealed that younger procedural age < 70 years (mean responder age, 69.7; mean failure age, 72.7; OR: 2.04, *p* < 0.01) and shorter pre‐procedural catheter duration (mean responder duration, 53.2; mean failure catheter duration, 198.7; OR: 1.004, *p* < 0.01) predicted success. Though only measured in PULSAR, higher BVE measured between postoperative days 3–7 (59%; mean failure voiding efficiency, 32%; OR: 1.02, *p* = 0.02) and as a continuous variable also predicted successful resolution of retention at 12 months post‐PUL (Figure 3). Stepwise multivariate regression analysis did not reveal interaction between any variables tested.

## 4 DISCUSSION

International clinical guidelines suggest that AUR be treated with immediate temporary urinary catheterization and the initiation of alpha‐blockers,^14^ a regimen that fails in approximately 50% of patients at 6 months.^15^ Consequently, men left catheterized are often referred to a specialist for surgical BPH treatment evaluation or added to long surgical waiting lists. Currently, the British Medical Association reports significant treatment backlogs and longer wait times for patients in the United Kingdom, with the largest number of patients in history awaiting BPH treatment and other consultant‐led elective care.^16^ These delays are not benign: Longer catheter duration is associated with repeated catheter/trial to void cycles (possibly facilitating ongoing bladder damage), increased urosepsis events, urethral injury and higher likelihood of failure once the intervention is attempted.^17^, ^18^ Up to half of all men with one episode of AUR will experience recurrent retention and 85% will proceed to surgical intervention within a year^5^; a timely and effective intervention to relieve their obstruction is essential.

PUL is a standardized minimally invasive procedure, taking on average less than 30 min, and may be performed as a day case (under 2‐h average hospital stay in the PULSAR study). Although patients had a mean retention duration of 6 months prior to PUL, 71% of subjects were catheter‐free at 1 month post‐PUL, rising to 73% at 12 months. Despite the inability to measure baseline urinary symptoms in catheterized patients, favourable urinary symptom outcomes emerged at the earliest timepoints (6 weeks post‐procedure) and continued to improve through study end at 12 months. QoL was substantially improved, surpassing those reported in previous PUL‐controlled clinical trials; this outcome also reflected in high patient satisfaction scores and likelihood of recommending the PUL procedure to a friend. The marked improvement in QoL score is likely due to the experience of being freed from a catheter in addition to achieving a similarly reduced level of LUTS to that seen in the LIFT trial. When PUL is performed as a day case, it also provides cost savings in comparison to TURP and HoLEP.^19^

### 4.1 PULSAR outcomes

This is the first time urodynamic outcomes have been reported in retention patients following PUL, demonstrating protection from bladder deterioration caused by prolonged obstruction, as well as a willingness by some BPH patients to undergo urodynamics. Furthermore, we have shown that in some AUR patients, PUL can restore bladder function to within normal urodynamic parameters. Overall, we see improvements in detrusor activity in patient populations with advanced disease, raising the possibility of even greater improvement in bladder function if PUL were introduced earlier in the disease process. In patients who remain obstructed or equivocal on uroflowmetry, QoL improvements (i.e., high catheter‐free rates and satisfaction with urinary symptoms) demonstrate that PUL may still meet patient goals. It has been shown that purely objective measures (such as Pdet@Qmax and BOOI) do not fully correlate with IPSS or QoL reporting and men may demonstrate equivocal or obstructed BOOI while remaining asymptomatic.^20^, ^21^

PULSAR patients returned quickly to their preoperative activity levels in just over a week (i.e., an average of 8 days) following PUL. Patients who were sexually active at baseline also preserved their pre‐existing erectile function after PUL. Although 100% of patients required alpha‐antagonist medications prior to PUL, 73% of these men were able to discontinue and remain off medication by study end. Perioperative adverse events were mild to moderate, with dysuria and haematuria the most commonly reported concerns; CTCAE Grade 3 or higher events all resolved within days; eight patients required BPH surgical intervention.

### 4.2 PULSAR in comparison with controlled and real‐world studies

In comparing PULSAR to LIFT, we found that IPSS and Qmax scores were similar at 12 months, and PULSAR QoL scores were significantly better at all timepoints, findings made even more noteworthy by the larger baseline prostate volumes in the PULSAR cohort. Sexual function remained stable in both PULSAR and LIFT groups through 12 months post‐procedure.

Outcomes in treating AUR patients were consistent in a real‐world setting. Catheter‐free rates were similar at 30 days and 12 months between PULSAR subjects and real‐world patients, and overall adverse event rates were not elevated in the real world. Furthermore, symptom response was comparable between settings despite PULSAR patient prostate volumes being significantly larger at baseline. The ability to wean AUR patients off catheter with PUL (71%–72% by 30 days; 73%–80% by 12 months) is reduced in comparison with 12‐month catheter‐free rates reported for TURP and PVP (79.1% and 88.7%, respectively).^22^ Another available MIST, water vapour thermal therapy (i.e., Rezum steam injection), has a similar reported success rate; the average post‐procedural catheter duration was approximately 1 month following Rezum.^23^, ^24^ This difference is likely because PUL mechanically opens the prostate, whereas steam ablation causes oedema and requires tissue resorption in order to take effect.^25^

In assessing baseline characteristics that may predict continued catheterization or retreatment at 12 months post‐PUL, univariate regression analysis of the RWRr group found that advancing age, higher baseline PVR and PSA, and a longer pre‐procedure catheter duration were associated with failure. Furthermore, early post‐TWOC BVE measurements were predictive of patients achieving catheter independence at 12 months with a 60% BVE cut‐off helpful in demonstrating a correlation with successful outcomes in this patient cohort. We calculated an OR of 1.026 that can be interpreted that for every percentage of BVE increase, the OR increased by a factor of 1.026. The construct of BVE within urodynamics is well founded but less is known about the predictive power in men undergoing bladder outlet surgery where higher cut‐offs have been proposed.^26^, ^27^

This prospective, non‐randomized study lacks a comparison against alpha‐blockers; however, the real‐world evidence of this small cohort establishes a jumping‐off point for further comparative studies. Furthermore, the study design and sample size were focused on elucidating the rate for when subjects could obtain catheter independence quickly. Further studies aimed at advancing long‐term outcomes following the PUL procedure in this patient population are warranted but currently beyond the scope of this manuscript.

There are an increasing number of minimally invasive surgical therapies available for BPH, with variable levels of published data supporting durability or effectiveness. PUL has been extensively researched across multiple clinical subgroups both in controlled and real‐world settings, providing robust insight into the benefits of adopting PUL in men with BPH. These studies reliably reproduce the positive findings from the original LIFT‐controlled study. By using demographic matching and filtering, one can draw comparisons between patients from non‐concomitant RCTs and large, real‐world studies. The PULSAR study represents the first controlled study to investigate the outcomes of a truly minimally invasive treatment such as PUL in this challenging patient cohort. The results detailed here allow us to provide contemporary data when counselling men in AUR who wish to undergo PUL surgery. The expanding body of evidence around PUL continues to demonstrate a consistent safety profile with excellent procedural outcomes that compare favourably with other BPH treatments, most of which offer shorter follow‐up durations, higher failure rates in the form of return to medication and a paucity of quality data in men with AUR.^28^

## 5 CONCLUSION

In this detailed analysis of PUL in patients with AUR, we show consistent and durable improvements in IPSS, QoL, Qmax and PVR through 12 months post‐procedure, with a favourable safety profile, erectile function preservation and rate of catheter independence. Seventy‐three percent of PULSAR patients achieved catheter independence and were free from surgical retreatment at 12 months post‐treatment. These findings are consistent with the LIFT pivotal trial outcomes and those seen in a large, RWR registry, in which 80% of retention patients achieved catheter independence, with younger age, lower baseline PVR and PSA and shorter pre‐procedural catheter duration influencing success. In PULSAR and real‐world retention groups, bladder voiding efficiencies following PUL may help to ascertain long‐term response to treatment. Collectively, these data may support the use of PUL as a day‐case treatment for BPH. This comparison of randomized controlled trial outcomes with real‐world data in large population provides a robust and clinically valuable view of the effectiveness and safety of PUL in treating AUR. Further, it supports PUL as a timely treatment for AUR in order to achieve optimal results and prevent further deterioration of bladder function. The ability to perform PUL as a day case procedure is particularly pertinent in healthcare systems with long waiting periods for hospital admissions and operating room time.

## AUTHOR CONTRIBUTIONS

Mark Rochester, Toby Page, Neil Barber, Rajesh Kavia, Nikesh Thiruchelvam and Oliver Kayes were investigators on the PULSAR feasibility clinical trial; Ruth Doherty performed all urodynamic assessments included in the trial. Steven Gange, Thomas Mueller, Gregg Eure and Peter Chin were investigators on the Real‐World Retrospective study of PUL. All PULSAR and Real‐World investigators had access to the clinical data tables; contributed to the study design and concept, analysis and interpretation of results; and provided critical review of the manuscript.

## CONFLICT OF INTEREST STATEMENT

Drs. Rochester, Barber, Gange, Mueller, Eure, Chin and Kayes are Neotract Inc./Teleflex consultants.
